# Sacubitril/Valsartan Alleviates Experimental Autoimmune Myocarditis by Inhibiting Th17 Cell Differentiation Independently of the NLRP3 Inflammasome Pathway

**DOI:** 10.3389/fphar.2021.727838

**Published:** 2021-09-15

**Authors:** Wei Liang, Bai-Kang Xie, Pei-Wu Ding, Min Wang, Jing Yuan, Xiang Cheng, Yu-Hua Liao, Miao Yu

**Affiliations:** Department of Cardiology, Union Hospital, Tongji Medical College, Huazhong University of Science and Technology, Wuhan, China

**Keywords:** sacubitril/valsartan, Th17 cell differentiation, NLRP3 inflammasome, myocarditis, NF-κB p65

## Abstract

Sacubitril/valsartan (Sac/Val) is a recently approved drug that is commonly used for treatment of heart failure. Several studies indicated that Sac/Val also regulated the secretion of inflammatory factors. However, the effect and mechanism of this drug modulation of inflammatory immune responses are uncertain. In this study, an experimental autoimmune myocarditis (EAM) mouse model was established by injection of α-myosin-heavy chain peptides. The effect of oral Sac/Val on EAM was evaluated by histological staining of heart tissues, measurements of cardiac troponin T and inflammatory markers (IL-6 and hsCRP). The effects of Sac/Val on NLRP3 inflammasome activation and Th1/Th17 cell differentiation were also determined. To further explore the signaling pathways, the expressions of cardiac soluble guanylyl cyclase (sGC) and NF-κB p65 were investigated. The results showed that Sac/Val downregulated the inflammatory response and attenuated the severity of EAM, but did not influence NLRP3 inflammasomes activation. Moreover, Sac/Val treatment inhibited cardiac Th17 cell differentiation, and this might be associated with sGC/NF-κB p65 signaling pathway. These findings indicate the potential use of Sac/Val for treatment of myocarditis.

## Introduction

Myocarditis is an inflammatory disease of the myocardium whose manifestations range from subclinical disease to heart failure, cardiogenic shock, arrhythmias, and sudden death (Caforio et al., 2013). Myocarditis is mainly caused by viral or bacterial infections and autoimmune disorders (Kindermann et al., 2012). Multiple innate and adaptive immune responses such as activation of NLRP3 inflammasomes and Th1/Th17 cell differentiation occur during myocarditis (Yuan et al., 2010; Yu et al., 2013; Tschöpe et al., 2017; Wang et al., 2019; Zarak-Crnkovic et al., 2019). Sacubitril/valsartan (Sac/Val) is an angiotensin receptor-neprilysin inhibitor (ARNI) that reduces the risk of death and hospitalization of patients with heart failure, and researches show that it is superior to angiotensin-converting enzyme inhibitor/angiotensin receptor blocker (ACEI/ARB) in alleviating heart failure (Lefer and Sharp, 2018; Velazquez et al., 2019). Moreover, an animal experiment revealed that Sac/Val treatment improved the prognosis of acute myocardial infarction (AMI) by suppressing the expression of IL-1β and IL-6, demonstrating the anti-inflammatory effect of this drug (Ishii et al., 2017). However, its effect in inflammatory diseases and the mechanism underling its anti-inflammatory pathways are not well understood.

Herein, we advanced this line of research using experimental autoimmune myocarditis (EAM) mouse model, which was considered as a useful tool to study myocarditis and even a large group of inflammatory diseases (Hoetzenecker et al., 2015). And we investigated whether and how Sac/Val would regulate inflammatory response compared with the ARB valsartan in EAM.

## Materials and Methods

### Materials

Complete Freund’s adjuvant (CFA) was bought from Sigma (Shanghai, China). Sac/Val and valsartan were purchased from Novartis Pharma AG (Basel, Switzerland). Antibodies against IFN-γ (Cat# sc-8423, RRID: AB_627,778), IL-1β, IL-17 (Cat# sc-374218, RRID: AB_10988239), and β-actin were bought from Santa Cruz (Dallas, USA). Anti-IL-18 antibody was purchased from Arigobio (Hsinchu City, Taiwan). The IL-6 ELISA kit (Cat# M6000B, RRID: AB_2877063) and the hsCRP ELISA kit were purchased from R andD (Minneapolis, USA). The IFN-γ and IL-17 ELISA kits were purchased from Neobioscience (Shenzhen, China). The cTnT quantitative rapid assay kit and collagenase B were bought from Roche Diagnostics (Shanghai, China). The total protein extraction kit, super ECL reagent, and antibody against ROR-γt (Cat# 14-6988-80, RRID: AB_1311291) were bought from Thermo Fisher Scientific Inc. (Hanover Park, USA). Antibodies against NLRP3 (Cat# AG-20B-0014, RRID: AB_2490202), ASC (Cat# AG-25B-0006, RRID: AB_2490440) and caspase-1 (Cat# AG-20B-0042, RRID: AB_2490248) were purchased from AdipoGen. Antibodies against T-bet, sGC-α1, sGC-β1, total-NF-κB p65 and phospho-NF-κB p65 were purchased from ImmunoWay (Plano, United States). FITC-anti-mouse CD4, PE-anti-mouse IFN-γ and allophycocyanin-anti-mouse IL-17 were purchased from eBioscience.

### Mice

All animal experimental procedures were approved by the Institutional Animal Care and Use Committee at Tongji Medical College, Huazhong University of Science and Technology. The animal experiments were performed according to the guidelines of the National Institutes of Health Guide for the Care and Use of Laboratory Animals, and the procedures were as humane as possible. Six weeks-old male BALB/c mice (MGI Cat# 2161059, RRID: MGI: 2161059) were purchased from Huazhong Agricultural University (Hubei province, China) and were reared at Tongji Medical College of Huazhong University of Science and Technology in a pathogen-free room under a 12 h light/12 h dark environment at 23°C.

EAM was induced using subcutaneous injection of 100 μg of MyHC-α peptide (Ac-SLKLMATLFSTYASAD-OH) emulsified with CFA in a 1:1 ratio (Shanghai, China). The drug was administered on days 0 and 7 as previously described (Eriksson et al., 2003). The mice were then randomly divided into four groups, each with six mice: (i) the “Control group” received oral gavage of corn oil (1 ml kg^−1^·day^−1^); (ii) the “EAM group” received subcutaneous injections of MyHC-α and oral gavage of corn oil; (iii) the “Valsartan group” received subcutaneous injections of MyHC-α and oral gavage of valsartan (10 mg kg^−1^·day^−1^) dissolved in corn oil; (iv) the “Sac/Val group” received subcutaneous injections of MyHC-α and oral gavage of Sac/Val (20 mg kg^−1^·day^−1^) dissolved in corn oil. Sac/val dosage was based on the maximum dose that did not lower baseline blood pressure (Ishii et al., 2017). The hearts and samples of orbital blood were collected on day 21 for further assessments prior to euthanize the mice. All operations were performed under anesthesia induced by intraperitoneal injection of ketamine (150 mg kg^−1^).

### Histopathology

The ventricular tissues of the hearts were fixed in 10% phosphate-buffered formalin, cut in to thin 5-mm sections before embedding in paraffin. The tissue sections were further cut longitudinally and stained using H&E. The severity of myocardial injury based on the percentage of heart inflammation was determined using a microscopic eyepiece grid ( × 200). The degree of myocardial injury was gradated by histopathological microscopic approximation of the percent area of myocardium infiltrated with mononuclear cells. Details were as follows; grade-0, no injury; grade-1: <25%; grade-2: 25–50%; grade-3: 50–75% and grade-4: >75% (Nishio et al., 1999). Assessments were performed by two independent investigators who were blinded to group allocations. And four images covering nearly the entire section per sample were analyzed for histopathology.

### Immunohistochemistry Analysis

Detection and quantification of IL-1β, IL-18, IFN-γ, and IL-17 on the paraffin-embedded ventricular tissues were performed using immunohistochemistry (IHC) analysis. The antibodies used included anti-IL-1β antibody (1:200) purchased from Santa Cruz Biotechnology (CA, United States), anti-IL-18 antibody (1:200) purchased from Arigobio (Hsinchu, Taiwan), anti-IFN-γ antibody (1:200) purchased from Santa Cruz Biotechnology (Cat# sc-8423, RRID: AB_627,778), and anti-IL-17 antibody (1:200) purchased from Santa Cruz Biotechnology (Cat# sc-374218). Images of the stained sections were analyzed using a Leica DZ 2000 LED microscope purchased from Leica Microsystems (Wetzlar, Germany). Photos of the stained tissues were captured at ×400 magnification. The experiments and interpretations were all blinded.

### Elisa

The levels of serum hsCRP and IL-6 were measured using hsCRP and IL-6 ELISA kits (R&D), according to the manufacturer’s instructions. The minimum detectable levels were 0.006 ng mL^−1^ for hsCRP and 1.6 pg mL^−1^ for IL-6. The levels of serum IFN-γ and IL-17 were measured using IFN-γ and IL-17 ELISA kits (Neobioscience), according to the manufacturer’s instructions. The minimum detectable levels were 3.9 pg mL^−1^ for IFN-γ and 0.78 pg mL^−1^ for IL-17. All the experiments were performed in triplicate. The level of Serum cardiac troponin T (cTnT) was measured using a rapid assay kit from Roche Diagnostics (Shanghai, China) as previously described by Metzler (Metzler et al., 2001). The experiment was also performed in triplicate.

### Western Blotting

Total proteins in myocardial tissues were extracted using a total protein extraction kit purchased from Thermo Fisher Scientific (MA, United States). Thereafter, 40 μg of the proteins were separated using 8% or 13% SDS-PAGE electro-transferred onto polyvinylidene fluoride (PVDF) membranes and blocked for 3 h in TBST supplemented with 5% skim milk. After incubation at 4°C overnight with anti-NLRP3 antibody (1 μg mL^−1^; AdipoGen, RRID: AB_2490202), anti-ASC antibody (1:1,000; AdipoGen), anti-caspase-1 antibody (1 μg mL^−1^; AdipoGen, RRID: AB_2490248), anti-T-bet antibody (1:1,000; ImmunoWay), anti-ROR-γt antibody (5 μg mL^−1^, Thermo Fisher Scientific, RRID: AB_1311291), anti-sGC-α1 antibody (1:1,000; ImmunoWay), anti-sGC-β1 antibody (1:1,000; ImmunoWay), anti-phospho-NF-κB p65 antibody (1:1,000; ImmunoWay), anti-total-NF-κB p65 antibody (1:1,000; ImmunoWay) and anti-β-actin antibody (1:1,000) purchased from Santa Cruz Biotechnology (CA, United States), the membranes were rinsed three times using PBS before the second incubation for 2 h at room temperature with secondary anti-rabbit or anti-mouse HRP-conjugated IgG (1:20,000) purchased from Jackson ImmunoResearch. The bands for target proteins were detected using the super enhanced chemiluminescence (ECL) reagent purchased from Thermo Fisher Scientific.

### Flow Cytometry

Hearts from the experimental mice were cut into small 1 mm^3^ cuboidal sections and digested for four times at 37°C in a water bath using 0.1% collagenase B (Roche Diagnostics GmbH) as previously described (Liao et al., 2012). Each round of digestion lasted 6 min. The cell suspensions were spread over a Ficoll-Hypaque density gradient solution to extract mononuclear cells. After three washings with PBS, the mononuclear cells were resuspended in PBS at cell density of 1.5 × 10^6^ mL^−1^. The cells were stimulated after culturing at 37˚C for 4 h under 5% CO_2_ in RPMI 1640 medium supplemented with PMA (20 ng mL^−1^), ionomycin (1 mg mL^−1^) and monensin (2 mmol L^−1^) in a 24-well culture plate. Thereafter, the cells were stained with FITC-labeled anti-mouse CD4 antibody (eBioscience), rinsed, fixed and permeabilizing using corresponding reagents. The cells were then stained using PE-labeled anti-mouse IFN-γ and allophycocyanin-labeled anti-mouse IL-17 A antibodies (eBioscience) following 30 min incubation at 4˚C. After rinsing, the percentages of CD4/IFN-γ-positive cells and CD4/IL-17 A-positive cells were measured using FACScalibur flow cytometry (BD, RRID: SCR_000401). Controls were stained with Ab-conjugated matched isotopes controls. The details of the isotype antibodies are as follow: Rat IgG2b kappa-FITC (eB149/10H5), Rat IgG1 kappa-PE (eBRG1) and Rat IgG2a kappa-APC (eBR2a) (eBioscience). The data was analyzed using CellQuest software (BD CellQuest Pro, RRID: SCR_014489Cite properly), and the minimum number of 5,000 events were collected and analyzed.

### Statistical Analysis

The data are expressed as means ± SEM. Power analyses were conducted and a sample of six mice per group attained >90% power at a 0.05 significance level for each experiment. Differences between groups were assessed using one-way ANOVA. Tukey’s post hoc test was also performed if the F value were significant and there was no significant heterogeneity between variances. The data was analyzed using SPSS V.22. A two-tailed *p* < 0.05 was considered statistically significant.

## Results

### Sac/Val Attenuated the Severity of EAM

**FIGURE 1:**
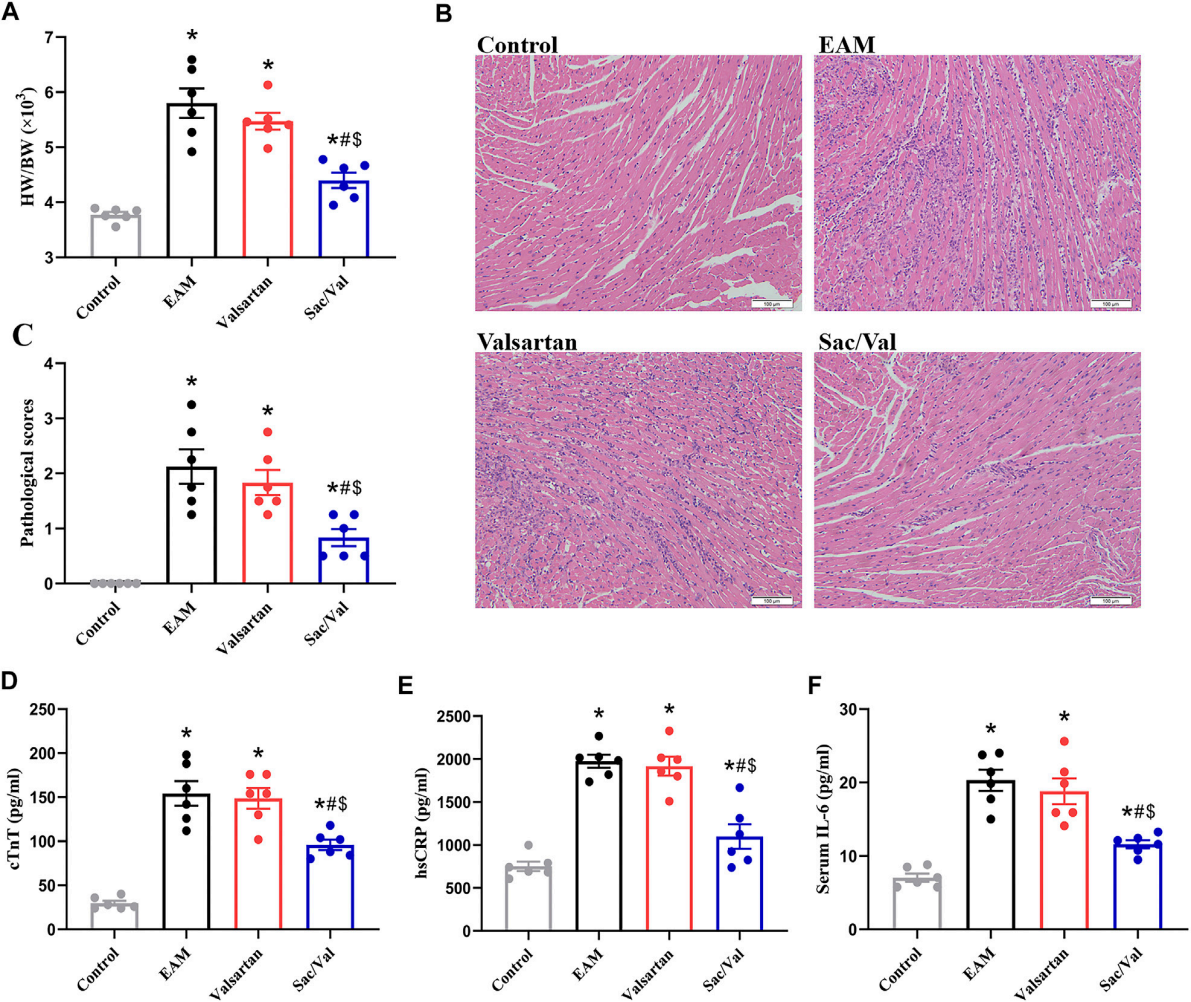
Sac/Val attenuated the severity of EAM. **(A)** HW/BW in each treatment group. **(B)** Typical histopathologic pictures of myocardial tissues (H and E staining, × 200). **(C)** The statistical graph of pathological scores in each treatment group. **(D)** Levels of serum cTnT in each treatment group. **(E)** Levels of serum hsCRP in each treatment group. **(F)** Levels of serum IL-6 in each treatment group. All experiments had six mice per group and data were expressed as means ± SEM and analysed by one-way ANOVA. ^*^ *p* < 0.05 *vs.* Control group; ^#^ *p* < 0.05 *vs.* EAM group; ^$^ *p* < 0.05 *vs.* Valsartan group.

The EAM, Valsartan and Sac/Val groups displayed higher heart weight/body weight ratio (HW/BW), pathological scores of heart sections and cTnT levels, relative to the Control group (*p* < 0.05). However, the above parameters were lower in the Sac/Val group, relative to both EAM and Valsartan groups (*p* < 0.05). There were no significant differences in the above parameters between the EAM and Valsartan groups (*p* > 0.05) (Figures 1A–D).

HsCRP and IL-6 are common inflammatory markers. Both serum hsCRP and IL-6 levels were significantly higher in the EAM, Valsartan and Sac/Val groups, relative to the Control group (*p* < 0.05). However, the levels of serum hsCRP and IL-6 in the Sac/Val group were lower than those in the EAM and Valsartan groups (*p* < 0.05), but there were no differences in these proteins between the EAM and Valsartan groups (*p* > 0.05) (Figures 1E,F). These results showed that Sac/Val treatment downregulated the expression of inflammatory factors, attenuated the severity of EAM.

### Sac/Val had No Effect on Activation of NLRP3 Inflammasomes

**FIGURE 2:**
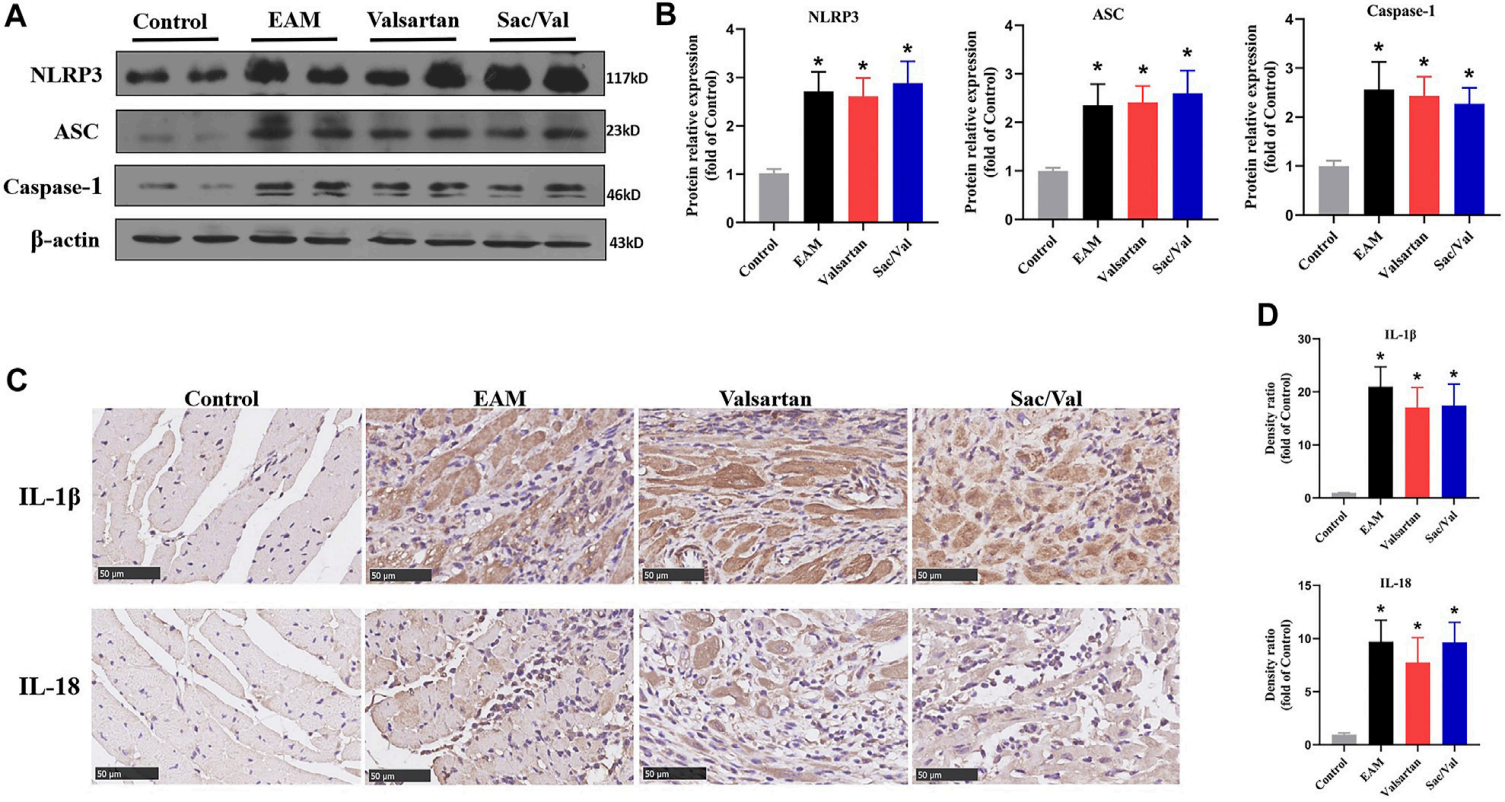
Sac/Val had no effect on activation of NLRP3 inflammasomes. **(A)** Representative western blotting results for cardiac NLRP3, ASC, and Caspase-1. **(B)** Statistical analysis of the western blotting results. **(C)** Representative IHC images of cardiac IL-1β and IL-18 (magnification × 400). **(D)** Statistical analysis of the IHC results. All experiments had six mice per group and data were expressed as means ± SEM and analysed by one-way ANOVA. ^*^ *p* < 0.05 *vs.* Control group.

We measured the expressions of three NLRP3 inflammasome components (NLRP3, ASC, and Caspase-1) and their downstream cytokines (IL-1β, IL-18) in myocardial tissue. Western blot and IHC revealed that all the above proteins were over-expressed in the EAM, Valsartan and Sac/Val groups, relative to the Control group (*p* < 0.05). However, there were no differences in the expression of NLRP3, ASC, Caspase-1, IL-1β and IL-18 among the EAM, Valsartan and Sac/Val groups (*p* > 0.05) (Figure 2), suggesting that Sac/Val treatment had no effect on the activation of NLRP3 inflammasomes during EAM.

### Sac/Val Inhibited the Differentiation of Th17 Cells in Myocardial Tissue

**FIGURE 3:**
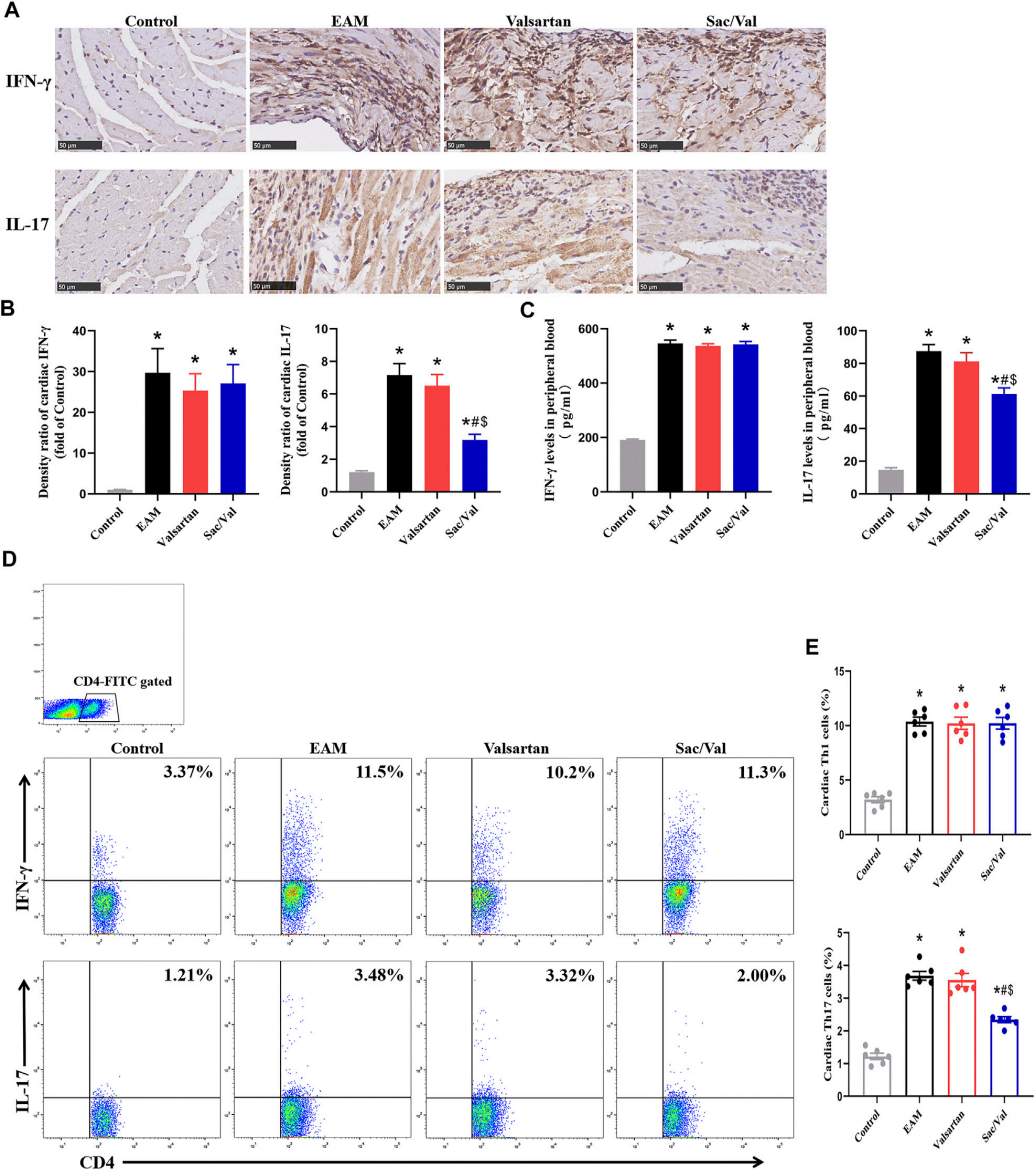
Sac/Val inhibited the differentiation of Th17 cells in myocardial tissue. **(A)** Representative IHC images of cardiac IFN-γ and IL-17 (magnification × 400). **(B)** Semiquantitative analysis of the IHC results. **(C)** Levels of peripheral blood IFN-γ and IL-17 in each treatment group. **(D)** Representative flow cytometry images of Th1 (IFN-γ^+^) and Th17 (IL-17^+^) cells gated on CD4^+^ T cells. **(E)** Statistical analysis of the flow cytometry results. All experiments had six mice per group and data were expressed as means ± SEM and analysed by one-way ANOVA. ^*^ *p* < 0.05 versus Control group; ^#^ *p* < 0.05 *vs.* EAM group; ^$^ *p* < 0.05 *vs.* Valsartan group.

There were higher expressions of IFN-γ and IL-17 in both myocardial tissue and peripheral blood in the EAM, Valsartan and Sac/Val groups, relative to the Control group (*p* < 0.05). Moreover, the levels of cardiac and peripheral blood IL-17 were lower in the Sac/Val group than those in EAM and Valsartan groups (*p* < 0.05), but there was no significant difference in the expressions of IL-17 between the EAM and Valsartan groups (*p* > 0.05). Notably, there was no significant difference in IFN-γ levels in both myocardial tissue and peripheral blood among the EAM, Valsartan and Sac/Val groups (*p* > 0.05) (Figures 3A–C).

There were significantly higher proportions of cardiac Th1 and Th17 cells in the EAM, Valsartan and Sac/Val groups, relative to the Control group (*p* < 0.05). Furthermore, the proportion of Th17 cells in myocardial tissue of the Sac/Val group was significantly lower than those of EAM and Valsartan groups (*p* < 0.05). However, there was no significant difference in the proportion of Th17 cells between the EAM and Valsartan groups (*p* > 0.05). Also, there were no differences in the proportion of cardiac Th1 cells among the EAM, Valsartan and Sac/Val groups (*p* > 0.05) (Figures 3D,E). Overall, results showed that Sac/Val treatment inhibited the differentiation of cardiac Th17 cells.

### Sac/Val Lowered Levels of RORγt in Myocardial Tissue

**FIGURE 4:**
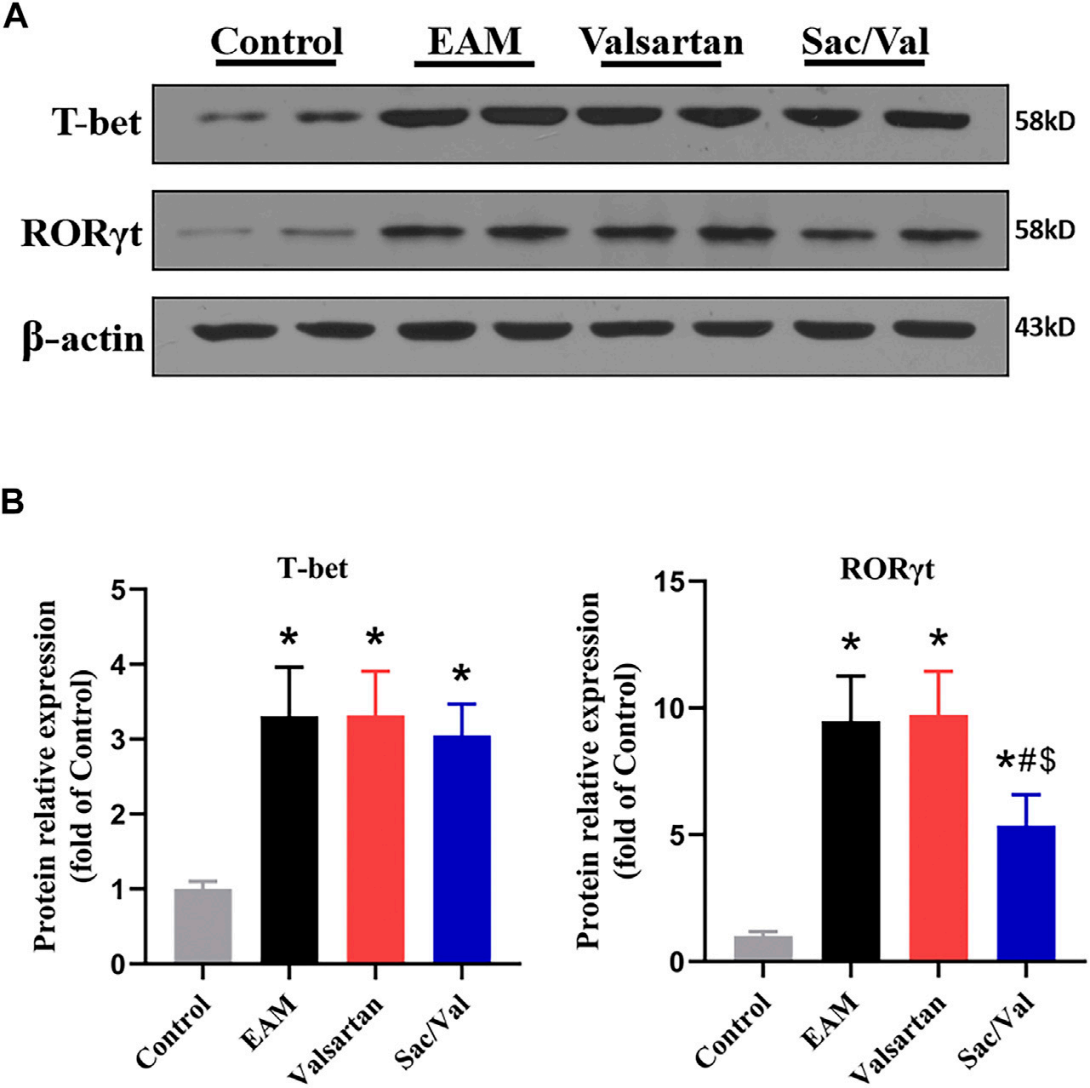
Sac/Val lowered levels of RORγt in myocardial tissue. **(A)** Representative western blotting results for cardiac T-bet and RORγt. **(B)** The statistical graph of the western blotting results. All experiments had six mice per group and data were expressed as means ± SEM and analysed by one-way ANOVA. ^*^ *p* < 0.05 *vs.* Control group; ^#^ *p* < 0.05 *vs.* EAM group; ^$^ *p* < 0.05 *vs.* Valsartan group.

T-bet and RORγt are respectively the key transcription factor for differentiation of Th1 and Th17 cells. We found the levels of cardiac T-bet and RORγt were significantly higher in the EAM, Valsartan and Sac/Val groups, relative to the Control group (*p* < 0.05). However, the levels of cardiac RORγt were lower in the Sac/Val group than those in the EAM and Valsartan groups (*p* < 0.05), but there was no significant difference in RORγt expression between the EAM and Valsartan groups (*p* > 0.05). In contrast, there was no significant difference in T-bet expression across the EAM, Valsartan and Sac/Val groups (*p* > 0.05) (Figure 4). These findings validated the inhibitory property of Sac/Val against RORγt, the key signal transduction protein for Th17 cell differentiation.

### sGC and NF-κB p65 Functioned as Signaling Molecules

**FIGURE 5:**
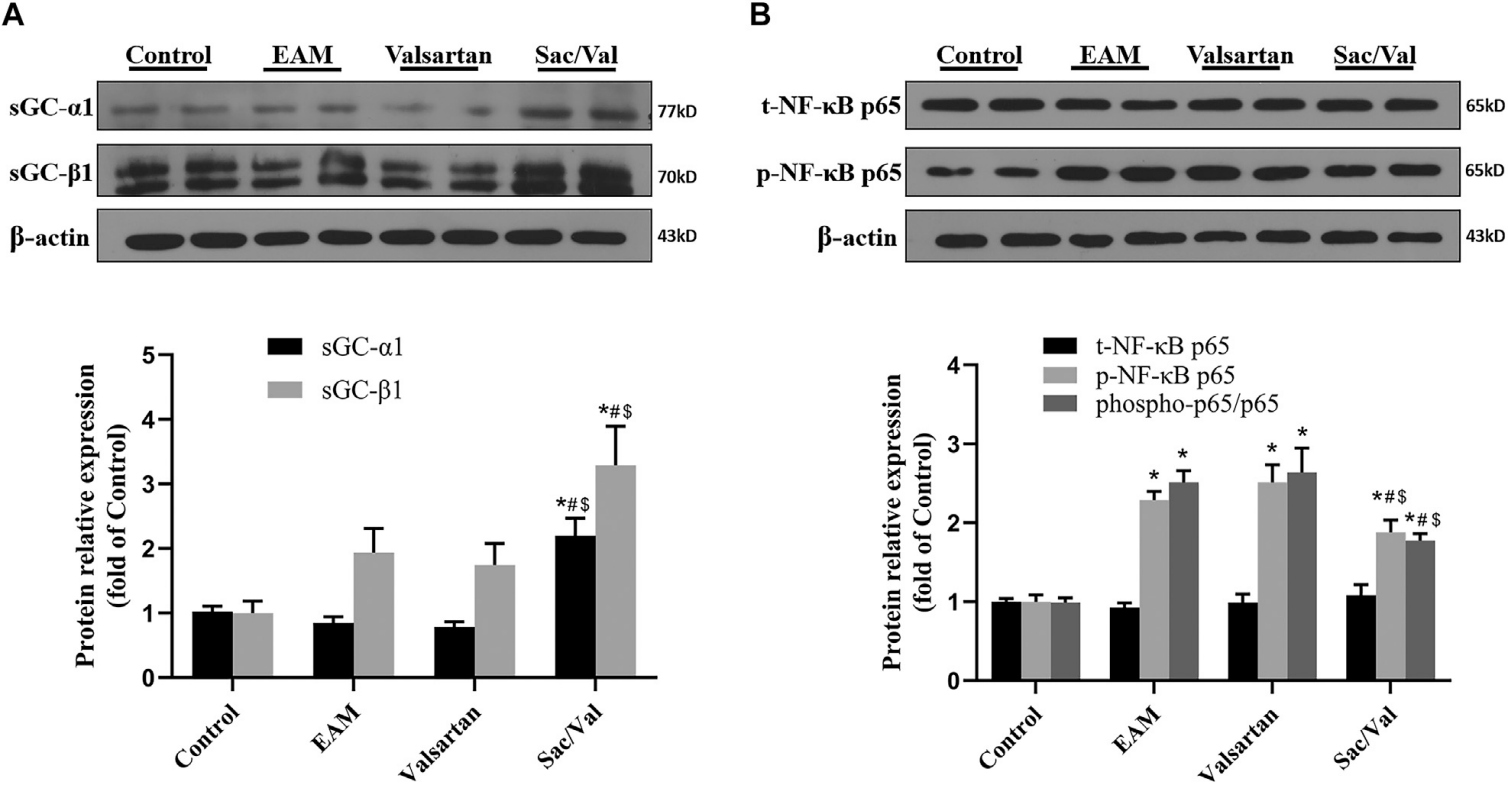
sGC and NF-κB p65 functioned as signaling molecules. **(A)** Representative western blotting images and statistical analysis for cardiac sGC-α1 and sGC-β1. **(B)** Representative western blotting images and statistical analysis for cardiac phospho-/total-NF-κB p65 signaling pathway. All experiments had six mice per group and data were expressed as means ± SEM and analysed by one-way ANOVA. ^*^ *p* < 0.05 *vs.* Control group; ^#^ *p* < 0.05 *vs.* EAM group; ^$^ *p* < 0.05 *vs.* Valsartan group.

The expressions of sGC-α1 and sGC-β1 were higher in myocardial tissue of the Sac/Val group than those in the Control, EAM and Valsartan groups (*p* < 0.05), but there was no significant difference in sGC expression among the Control, EAM and Valsartan groups (*p* > 0.05) (Figure 5A). The level of phospho-p65 was lower in the Sac/Val group, relative to those of EAM and Valsartan groups (*p* < 0.05), but there was no difference in phospho-p65 levels between the EAM and Valsartan groups (*p* > 0.05) (Figure 5B). These findings suggested that Sac/Val modulated inflammation associated with sGC and NF-κB p65.

## Discussion

We found Sac/Val modulated inflammatory damage in EAM by inhibiting the differentiation of Th17 cells probably via the sGC/NF-κB p65 signaling pathway, and this effect was independent of the NLRP3 inflammasome activation.

Sac/Val simultaneously suppresses the functions of neprilysin and ang II receptors, thus can provides clinical benefits to patients with heart failure (Ruilope et al., 2010; Mcmurray et al., 2014). According to previous studies, compared with the usage of valsartan alone, Sac/Val treatment downregulated the expression of inflammatory factors in apoE^−/−^ mice (Zhang et al., 2019). And Sac/Val is also superior to enalapril with regard to improving survival by inhibiting the acute phase of the post-AMI inflammatory response (Ishii et al., 2017). These studies suggest that Sac/Val is a potential therapeutic option against inflammatory heart diseases. Herein, we found Sac/Val treatment improved the ratio of HW/BW, serum level of cTnT and pathological scores of heart tissues, all associated with the severity of myocarditis. Moreover, Sac/Val treatment also down-regulated the expression of hsCRP and IL-6, both associated with the acute phase of inflammation. These findings indicated that Sac/Val had potent anti-inflammation effects in EAM.

Activation of NLRP3 inflammasomes is an initial step that led to inflammation (Chen and Nuñez, 2010; Duewell et al., 2010). NLRP3 inflammasomes are predominantly activated in innate immune cells such as macrophages, and regulate the activation of caspase-1, further induce the expression of IL-1β and IL-18 (Wen et al., 2013). IL-1β promotes the differentiation of naïve T cells into Th-17 subtype, and IL-18 increases the expression of IFN-γ by Th1 cells (Xu et al., 1998; Acosta-Rodriguez et al., 2007; Evavold and Kagan, 2018). Excessive activation of NLRP3 inflammasome has been implicated in the initial phase of numerous autoinflammatory and autoimmune diseases including EAM (Strowig et al., 2012; Chen et al., 2020). We observed overactivation of NLRP3 inflammasomes in EAM mice, which was consistent with previous findings. However, Sac/Val treatment had no significant effect on the expressions of NLRP3 inflammasome or its downstream cytokines (IL-1β and IL-18) in the heart. Accordingly, we speculated that during myocarditis, the anti-inflammatory effects of Sac/Val treatment were more pronounced in the downstream adaptive immune response.

T cells are the main players in adaptive immunity, they are also important participant in inflammatory diseases. Th1 and Th17 cells regulate acute inflammatory responses and play critical roles in the development of myocarditis (Eriksson et al., 2001; Cooper, 2009; Yuan et al., 2010; Yamashita et al., 2011; Yu et al., 2013; Yuan et al., 2014). Mechanistically, Th1 cells mainly secrete IFN-γ, which exacerbates the inflammatory response and promote myocardial infarction in autoimmune myocarditis (Eriksson et al., 2001; Nishikubo et al., 2007). Also, Th17 cells can infiltrate the myocardium, where they secrete inflammatory factors such as IL-17 (Yamashita et al., 2011; Yu et al., 2013; Yuan et al., 2014). In addition to its proinflammatory effects, IL-17 worsens myocarditis by increasing the generation of autoantibodies against heart proteins (Yuan et al., 2010). In this study, we further revealed that Sac/Val treatment downregulated the levels of IL-17 in both myocardial tissue and peripheral blood, and decreased the infiltration of Th17 in myocardial tissue of mice with myocarditis. Furthermore, we found Sac/Val suppressed the expression of RORγt, the key signal transduction protein that mediated differentiation of Th17. However, Sac/Val had no effects on the expression of IFN-γ and T-bet as well as the infiltration of Th1 cells. Thus, Sac/Val inhibited polarization of naïve CD4^+^ T cells to Th17 phenotype.

Taken together, Sac/Val exerts its anti-inflammatory effect against myocarditis by inhibiting the differentiation of Th17 cell, independently of NLRP3 inflammasome pathway. Furthermore, we try to investigate the underlying mechanisms of its effect on inhibiting Th17 differentiation. Compared to valsartan, it is well known that Sac/Val treatment increases levels of natriuretic peptides (NPs) by suppressing the expression of neprilysin, further activates natriuretic peptide receptors (NPRs, also known as particulate GC) to generate cyclic guanosine monophosphate (cGMP) (Levin et al., 1998; Nishikimi et al., 2006; von Lueder et al., 2013; Mcmurray et al., 2014). In this study, we did not focus on this completely confirmed signaling pathway. Instead, we attempted to explore weather Sac/Val regulated the expression of sGC. Two forms of GCs exist in cells and tissues: cytosolic [NO-activated; soluble, (sGCs)] and membrane bound [NPs-activated; particulate, (pGCs)]. Although both pGC and sGC produce cGMP, their signaling pathways are relatively independent (Pyriochou and Papapetropoulos, 2005; Potter et al., 2006; Cerra and Pellegrino, 2007). Nevertheless, cross-talk between these two pathways remains unclear. Activation of NPR-C is demonstrated to stimulate NO production through coupling with endothelial nitric oxide synthase (eNOS) (Anand-Srivastava et al., 1996; Murthy et al., 1998); Sac/Val-mediated improvements in cardiac function are related to increases in NO bioavailability (Trivedi et al., 2018). The above studies suggestion that Sac/Val might activate sGC through increasing the production and bioavailability of NO. In agreement, we found Sac/Val treatment significantly increased the expression of α1 and β1 subunits of sGC in myocardial tissue.

NPRs are expressed on T cells (Boyd and Hugo, 1991; Vollmar et al., 1996; Mohapatra et al., 2004; Casserly et al., 2010). Several *in vitro* researches show that ANP stimulation can affect T cell function (Heim et al., 1996; Ma et al., 2013). After stimulating peripheral blood mononuclear cells (PBMCs) with ANP, only T lymphocyte existed inducing for GC, in contrast to other types of cells (Heim et al., 1996). ANP suppresses the differentiation of Th17 cells via the NPR-A/PI3K-Akt pathway in a dose-dependent (Ma et al., 2013), consistent with our findings. However, how Sac/Val modulates local T-cell immunity in the heart *in vivo* is not well understood. A few researches have revealed that NF-κB p65 is an important signaling pathway in local inflammatory environment of heart, where it induces differentiation of Th17 cells (Cheng et al., 2016; Sun et al., 2016). Another study has revealed that activated sGC inhibits the NF-κB p65 signaling pathway (Flores-Costa et al., 2020). Consequently, we evaluated the effect of activated cardiac NF-κB p65 in myocarditis disease condition. The results showed that Sac/Val treatment down-regulated the expression of phospho-p65, which corresponded with increase in sGC in myocardial tissue. Take together, Sac/Val treatment increases circulating NPs, and further improves the production and bioavailability of NO. sGC is the “receptor” for NO, and NO-mediated sGC activation inhibits the NF-κB p65 signaling pathway, and subsequently inhibits the differentiation of Th17 cells. As such, we speculated that Sac/Val inhibited Th17 cell differentiation in EAM probably through the sGC/NF-κB p65 signaling pathway.

To the best of our knowledge, this is the first study assessing the anti-inflammatory effects of Sac/Val under myocarditis condition. In general, Sac/Val alleviates myocarditis by inhibiting the differentiation of Th17 cells, and this process is independent of the NLRP3 inflammasome pathway. Particularly, it is possible that Sac/Val exerts its anti-inflammatory effect by activating sGC and inhibiting the NF-κB p65 signaling pathway. Thus, our findings provide evidence that supports the potential use of Sac/Val for the treatment of myocarditis. However, there were some limitations in this study. For example, there was a lack of functional assessment on the heart. Generally, in the acute phase of EAM (analyzed on day 21), the ratio of HW/BW, serum level of cTnT and inflammation pathological scores of heart tissues are frequently used to consider the severity of EAM. And the indicators of heart function start to change at week 4, and gradually develop into the chronic phase (analyzed between days 45 and 65) (Cruz-Adalia et al., 2010; Diny et al., 2017; Anzai et al., 2019). Here, we focused on the acute inflammation in EAM, so the functional assessment of heart was not performed. However, we will investigate the effect of Sac/Val on cardiac function in future works. And in the future, *in vitro* experiments are needed to detect the direct effects of Sac/Val or the substrates of neprilysin on various kinds of inflammatory cells infiltrated in myocardium, and to explore their mechanisms. Moreover, the mechanisms of Sac/Val activating sGC in EAM are currently not well understood, and in further research we will improve studies on this aspect, such as detection of NO and eNOS, and exploration of other undiscovered signaling molecules. Further clinical studies are also necessary to verify the efficacy of Sac/Val in myocarditis patients.

## Data Availability

The raw data supporting the conclusions of this article will be made available by the authors, without undue reservation.

## Abbreviations

ACEI, angiotensin converting-enzyme inhibitor; AMI, acute myocardial infarction; ARB, angiotensin receptor blocker; ARNI, angiotensin receptor-neprilysin inhibitor; cGMP, cyclic guanosine monophosphate; cTNT, cardiac troponin T; EAM, experimental autoimmune myocarditis; HW/BW, heart weight/body weight ratio; NP, natriuretic peptide; NPR, natriuretic peptide receptor; Sac/Val, sacubitril/valsartan; sGC, soluble guanylyl cyclase.
